# The London Homœopathic Hospital

**Published:** 1892-10-15

**Authors:** 


### HOSPITAL OPINION.

[Contributions critical, suggestive, controversial, and technical are invited for this column, which is open to everyone who has anything to say that is of practical value and interest.]

### THE LONDON HOMŒOPATHIC HOSPITAL.

Sir,—On my return to town my attention has been called to a statement in The Hospital as to the percentage of cost of administration at this hospital. Will your allow me to correct that statement by saying that the actual percentage of our cost of administration, as shown by our last balance-sheet—enclosed herewith—was 9·593 per cent. only. This balance-sheet is in the form recommended by the Committee of Hospital Managers, meeting at the request of the Hospital

Sunday Fund, is approved by the authorities of that Fund, and is certified, as you will see, by chartered accountants.

I have no knowledge of the method by which the computation you have quoted has been arrived at, but the enclosed balance-sheet will show the accuracy of my own figures.

You are also kind enough to suggest that the percentage of cost of administration which you have quoted may be due to circumstances under which our "struggle to live is far more severe than in the case of the larger and older general hospitals." The same balance-sheet will show at once, that while some activity may be practised at our hospital in obtaining funds from our supporters, the result can hardly be described as a "struggle to live." I, at least, know nothing of any such struggle. Neither do I think our financial position is more severe than that of the "larger and other general hospitals." For the first time during several years we have a deficit of £472 4s. 9d. on the yearly account —a deficit which is disappearing. The deficits of the "larger and older general hospitals" range, I believe, from £15,000 to £5,000, and I once computed that five of them laboured under a total aggregate deficiency of £50,000, which was then the amount of the actual debt of all the hospitals—including the "larger and older"—showing that the deficit of the entire metropolitan hospital system really consisted of the deficits of five of the "larger and older general hospitals." But anyone who will refer to my examination before my Lords of the Metropolitan Hospitals Committee will see that their Lordships were clearly not of opinion that the "struggle" at this hospital was essentially "severe," while a certain perceptible diminution of our Hospital Sunday Fund award this year is courteously explained by the authorities of the Fund on grounds which certainly do not comprise a "struggle to live."

I must admit that there are "larger" general hospitals, but as to the "older," may I point out that we have got beyond the stage of juvenescence. We are 43 years old. Charing Cross is only 58, University is only 59, St. Mary's is only 41, King's College is only 53, the Great Northern Central only 36 (all according to "Burdett's Hospital Annual"), and consequently we cannot yield much seniority to any but the great historic hospitals, St. Bartholomew's, St. Thomas's, Guy's, Middlesex, and St. George's.—Your obedient servant,

G. A. Cross, Secretary-Superintendent.

October 8th, 1892.

[We congratulate Mr. Cross upon the prosperity of his hospital which he so eloquently proves. Our suggestion was made as a testimony of our appreciation of the zeal and energy displayed in the administration. The statement that the percentage of the cost of administration to that of maintenance, as determined by the Committee of Distribution of the Hospital Sunday Fund, for the three years ending March 31st, 1891, is 18·16 is correct. The report Mr. Cross encloses is for the year ending March 31st, 1892, whereas the Hospital Sunday Fund figures are for an average of three years ending 1891. Further Mr. Cross refers to the cost of administration only, which is wholly different from the cost of administration to maintenance which we gave in the article referred to.—Ed. of The Hospital.]

